# Research on the impact of COVID-19 on the financial system: Evidence from Indonesia

**DOI:** 10.1371/journal.pone.0301123

**Published:** 2025-02-21

**Authors:** Darjana Darjana, Sudarso Kaderi Wiyono, Deddy Priatmodjo Koesrindartoto

**Affiliations:** 1 School of Business Management, Bandung Institute of Technology, Bandung, Indonesia; 2 Bank Indonesia, Jakarta, Indonesia; Sabaragamuwa University of Sri Lanka Faculty of Management Studies, SRI LANKA

## Abstract

This study investigates the financial system vulnerability and pandemic impact on the financial system of Indonesia in 2015–2021. Two kinds of analysis are involved, i.e.: cross-section analysis at a certain point in time and time-series analysis along a certain time-period. To measure the risks, the research employs risk profile and network analysis using the Financial Account Balance Sheet from Bank of Indonesia. The balance sheet of one institutional sector is associated to the balance sheet of another sector from both assets and liabilities, such as corporations, households, and the financial sectors. Afterwards, Difference-in-Differences (DID) method and the macroeconomic linkages exercises the COVID-19 impact on the financial system. The result shows as the key findings that the financial system is more vulnerable during the pandemic for banks, corporations, and governments, indicated by the more declining net-worth, along with the external balance slowdown. Furthermore, the macroeconomic linkages show that during the pandemic, the financial system is more reliant on the banks, government, and the Rest of the World (ROW).

## 1. Introduction

COVID-19 start spread to the whole world in early 2020. The pandemic has caused negative impacts on all aspects of everyday life. One affected aspect is finance, especially in its system vulnerability. Financial system vulnerabilities are circumstances that may place the financial system into the exposure of risks. According to Bank Indonesia Regulation (PBI) No. 16/11/PBI/2015 for Macroprudential Regulation and Supervision, the financial system includes the financial institutions, financial markets, financial infrastructure, non-financial companies, and households. They interact with each other through funding or providing economic financing [1–5]. Thus, financial system elements are financial institutions (including banks and nonbanks), financial markets and infrastructures, as well as non-financial institutions and households. However, there is not much research on evaluating the impact of the pandemic on financial system vulnerabilities regarding the risk profile analysis and empirical studies. This study fills the research gap through exploration on financial system vulnerabilities before and after the COVID-19 pandemic.

Covid-19 disrupted both directly the supply and demand side of the economy. Gopinath [6] revealed that the coronavirus epidemic involves both supply and demand shocks. Business disruptions have lowered production, creating shocks to supply. And consumers’ and businesses’ reluctance to spend has lowered demand. As well as Georgieva [7] noticed that this shock is somewhat unusual as it affects significant elements of both supply and demand. Supply will be disrupted due to morbidity and mortality, but also the containment efforts that restrict mobility and higher costs of doing business due to restricted supply chains and a tightening of credit. Demand will also fall due to higher uncertainty, increased precautionary behavior, containment efforts, and rising financial costs that reduce the ability to spend. These effects will spill over across borders.

The study employ data from the financial account balance sheet from Bank of Indonesia. The balance sheet of one institutional sector is associated to the balance sheet of another sector from both assets and liabilities, such as corporations, households, and the financial sectors. IMF has conducted analysis of institutional sector vulnerability to all sectors of the economy using The Balance Sheet Approach (BSA) method. Their data position of assets and liabilities of each sector is analyzed to measure economic risks in a country [8, 9]. The imbalance risks emerging from the institutional sectors can be disseminated to the financial system across various channels [10].

The research objectives of this study are as follows. Firstly, to identify and analyze the risks and the interlink (network) of institutional sectors in the financial system by utilizing National Financial Account Balance Sheet (NFABS) as a data framework in implementing Balance Sheet Approach (BSA). Secondly, to conduct an impact analysis of COVID-19 pandemic on the institutional sectors in the financial system using NFABS. This can be continued to sub-sector industries.

Here the research conducts two kinds of analysis, i.e.: cross-section analysis at a certain point in time and time-series analysis along a certain time-period. As the cross-section analysis, Risk Profile analysis and Network analysis are conducted to identify the vulnerability in each institutional sector in the financial system and interlink risks among them. Meanwhile, the Difference-in-Differences (DID) is applied as a time-series analysis to do an impact evaluation of COVID-19 pandemic on the vulnerability of financial system. Macroeconomic linkages explain more about the interconnectedness among the institutional sectors.

The risk-analysis results present the view of the financial system in the net-borrowing condition contributed by the negative net-worth. Network analysis discloses that the topology of the financial system network has no change during the pandemic, while in terms of liabilities/ assets corporations, households, and the Rest of the World (ROW) are the most vulnerable institution. DID results also reveal that all institutional sectors are affected except for corporations and the ROW, with varying impact on key sub-sector industries. Furthermore, the macroeconomic linkages show that the Indonesian financial system is more reliant on the banks, government, and the ROW during the pandemic than previous periods.

Combination of the three analytical methods namely risk profile analysis, network analysis and DID method, as well as connections of macroeconomic to assess the financial system vulnerability is the novelty for research contribution. This research can complement other related exercises such as bank stress tests conducted at the national level by central banks and supervisory authorities for the measurement of banks’ resilience. Applicable for the international institution such as the IMF and World Bank in which conducted Indonesia Financial System Assessment Program (FSAP) in 4–5 years regularly (the last IMF-WB FSAP in Indonesia was conducted on March 2018 and plan undertake in the post-COVID 2023).

## 2. Literature review and hypothesis

**Fig 1:**
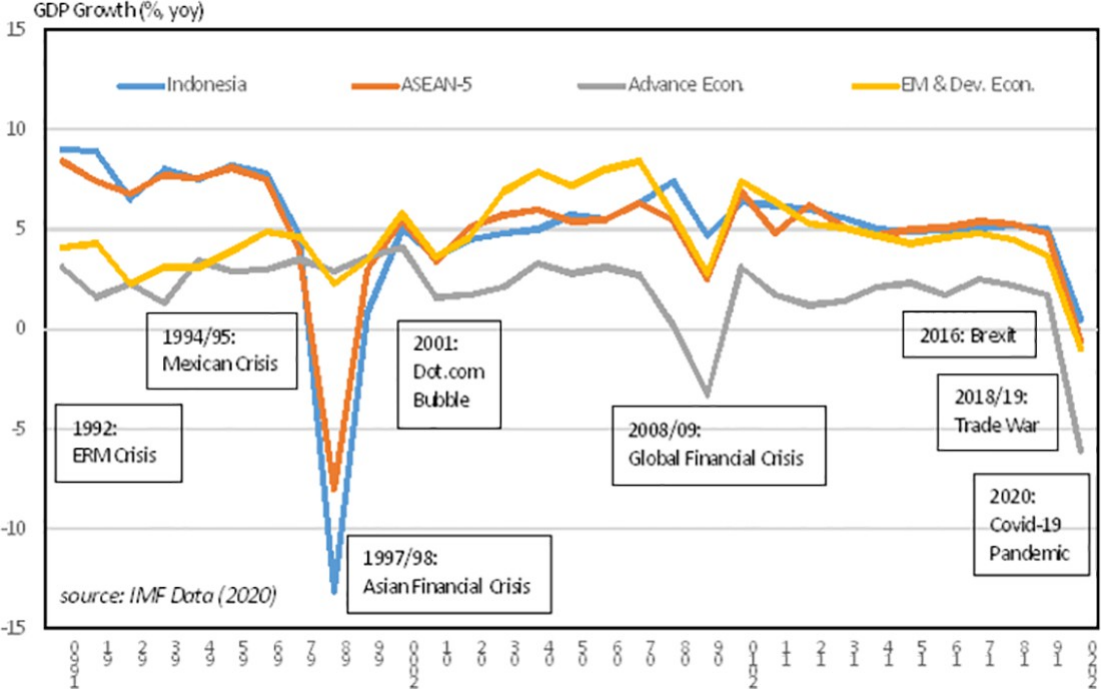
Black Swan event of crises.

The issue regarding financial system vulnerability is closely related to 2022 Nobel Prize for the Banks and Financial Crises Research by Ben S. Bernanke, Douglas W. Diamond, and Philip H. Dybvig. Our paper contributes to the literature of financial system vulnerabilities assessment using a certain point and time series analysis. Some other research utilizes statistical analysis such as probit model [11], vector error correction [12], and structural equation model [13]. Others explore collateral repo haircuts as well as the financial stress index regionally [14–17], propose a financial vulnerability indicator as composite indicators [18], or introduce a composite financial stability index and estimate monetary policy reaction functions using a nonlinear autoregressive model [19]. There is also research on new issues including the financial risk identification in big data era utilizing the emerging tools [20] and how green economy stabilizing the world financial system [21]. Fig 1 shows that the Covid-19 pandemic threaten global economics which includes the financial system stability of Indonesia.

Furthermore, the research adds literature on the financial system vulnerability related to the balance sheet approach and COVID-19. Some research evaluates the degree of household financial vulnerability [22–24], while others [25, 26] evaluate the COVID-19 pandemic’s impact on household finances or financial systemic risks.

The study also complements the implementation of the Difference-in-Differences method in literatures to analyze the financial stability or the influence of the COVID-19 pandemic on financial systems. Mishra and Dubey [27] utilize DID framework to identify the effect of their indices of financial and sector specific stability. Maiti [28] investigates how the COVID-19 pandemic affect the employees in the tourism and hospitality industry in India by the income and wages. Qadry et al. [29] investigate unpaid leave on the COVID-19 phenomenon in Pakistan related the pandemic into the psychology effects. Coad et al. [30] explore how the COVID-19 outbreak affected expectations for investment. The COVID-19 pandemic has negatively impacted on the South Asian’s bank financial performances especially in terms of profitability [31]. However, technological advancement has helped to maintain their financial performance during the pandemic. COVID-19 also has a significant negative impact on the stock market performance, i.e.: decrease the returns and increase the volatility [32]. Yan et al. [33] investigate how the banks’ contribution to systemic risk has been altered by the COVID-19 outbreak. Silva et al. [34] examine the interconnectivity effects on stock returns during the Global Financial Crisis using panel data by designing Difference-in-Differences (DID) econometric specifications. Wu [35] employs Difference-in-Differences method to investigate the effects of low odds/high payout of Illinois’ lottery incentive, as well as high odds/low payout of Wisconsin’s fixed incentive, on the county first-dose COVID-19 vaccination rates in 2021.

The study also enriches the literature of network analysis approach to measure financial systemic risk. Some literatures employ network analysis relating to complex interconnectedness and systemic risks during COVID-19 pandemic [36–39]. Ye and Li [40] discover that the network structure between public companies in China regarding its interconnectedness changed significantly after the COVID-19 pandemic. Baumöhl et al. [41] analyze the interconnectedness of the banking system and identify the contribution of each bank based on the topological network properties. Some research focus on cyber mapping of key financial and technology interconnections, network analysis, and stress testing [42], assess the key elements and applications of financial network resilience processing in financial regulation [43], present a network analysis model to explore the relationships before and during the pandemic crisis [44], or develop a bipartite financial network and its indicators in terms of the systemic risk [45]. Others consider bank networks and analyze characteristics as well as risk changes in different COVID-19 periods based on the network measurements [46–49].

Based on such related works, the hypothesis of this study is as follows. Using cross-section analysis at a certain point in time and time-series analysis, the study can identify financial system vulnerability and pandemic impact on the financial system of Indonesia.

## 3. Data and methodology

**Fig 2:**
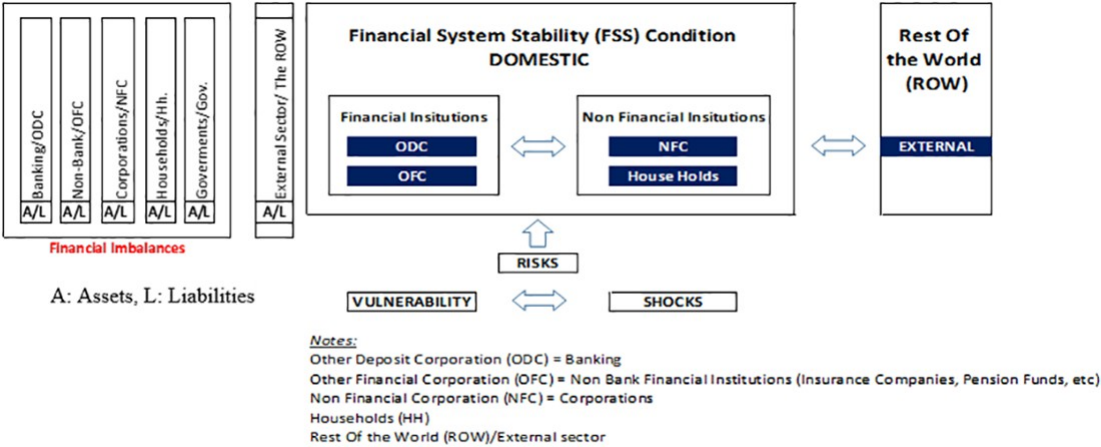
Institutional sectors in financial system and linkages.

According to Bank Indonesia, vulnerabilities can be identified through the behavior of each element in the financial system, such as measuring the performance and risks. The structure of financial system consists of 5–6 elements of the institutional sectors (see Fig 2), namely: Deposit Taking Institutions (Banking/ODCs), Non-Deposit Taking Institutions (Non-Banking/OFCs): insurances, pension funds, et cetera, Non-Bank Financial Institutions (Corporations/NFCs), Households (Hh), Government (Govt.), and External (Rest of The World/ROW). Institutional sector balance sheets provide a framework for comprehensive and in-depth analysis of risks and policies. Using the Financial Account Balance Sheet, the study conducts some exercises of the vulnerability indicated by financial imbalances.

### 3.1 Data

Quantitative research methods applied in the research with secondary data from official resources. The form of secondary data used in the research derived from multiple sources [50]: (1) Snapshot multiple source secondary data consists of data drawn from more than one source which relates to a single time, such as financial data and government publication; (2) Time-series multiple source secondary data are created by combining comparable variables, such as fiscal statistics (Ministry of Finance), monetary & external statistics (Bank Indonesia), and real sector statistics (Indonesia Statistics).

This research employ data ultimately from the national financial account balance sheet (NFABS) from Bank Indonesia. The balance sheet of one institutional sector is associated to the balance sheet of another sector from both assets and liabilities, such as corporations, households, government, rest of the world and the financial sectors. The net-worth quarterly series data of each institutional sector from NFABS since 2015: Q1 until 2021: Q4 (see Appendix). Then, the Current Account Balance (CAB) data with the same time horizon came from the Indonesia Financial Statistics (IFS) of Bank Indonesia. The data series utilized to risk profile and DID analysis of the COVID-19 impact on the financial system. Furthermore, the net-worth data from whom-to-whom liabilities positions of 2019: Q4, 2020: Q4, and 2021: Q4 exercised for network analysis. The last data, macroeconomic interlinkages derived from Indonesia Statistics, Ministry of Finance, and Bank Indonesia compiled 2018–2021 data in the IFS.

### 3.2 Methodology

This study analyzes the vulnerability of the financial system in Indonesia context. There are several analysis methods conducted, such as risk profile analysis, network analysis and macroeconomic linkages for cross section data, and DID method for time series data analysis.

#### 3.2.1 Risk profile analysis

Risk profile analysis measures vulnerability of the financial system in terms of the imbalance risks indicated by the net-borrowing or net-lending financial condition. This is such a risk monitoring tool of the financial system. Abubakar et al [51] use such analysis to identifies the vulnerability of household in Indonesia. Here, a sectoral risk profile analysis exercises the vulnerability of every institutional sector in the financial system.

The analysis calculates the inherent risk for the financial system. Following the Aggregate Demand (AD) function identity:

AD=Y=C+I+X–M (1)

Whereas Y: GDP, C: Consumption, I: Investment, X: Export, M: Import.

Afterwards, the Domestic Economy is separated from External Sectors to show interlink between them,

$$\left(\text{Y}–\text{C}\right)–\text{I}=\text{X}–\text{M}$$ (2)

$$\left(\text{Saving}\;\text{Investment}\;\text{Gap}\right)\text{S}–\text{I}=\text{X}–\text{M}\left(\text{Current}\;\text{Account}\;\text{Balance}\right)$$ (3)

$$\left(\text{Net}\;\text{Borrowing}\right)\text{S}<\text{I}=\text{X}<\text{M}\left(\text{Current}\;\text{Account}\;\text{Deficit}\right)$$ (4)

Indonesia’s external sectors show a deficit in current account balances (Current Account Deficit). It reflects the net borrowing condition in the domestic economy due to the negative saving investment gap. The net borrowing reveals the imbalance risks of the financial system. This condition can be contributed by the negative net-worth of several institutional sectors in the financial system.

#### 3.2.2 Network analysis

Here, the study discusses network analysis as an approach to modeling data in the financial system. This analysis can be used to describe the interlink among institutional sectors in the financial system. The nodes represent institutional sectors while the links represent the liabilities/ assets, which fall into debt financial network category [52]. Each node has a net worth value as calculated using the formula in Eq (5). Following are the main network measures that are useful for describing the overall structure of the network and characterising the position of a specific node in the network. The network statistic measurements include eigenvector centrality, weighted degree, and clustering coefficient. Eigenvector centralities enable us to identify the most important node within the network. The weighted degree of a node—as the sum of weights that assigned to incident links—shows the node strength. The study uses clustering coefficient to know how a node is embedded in the network structure. Afterwards, the research proceeds with risk analysis to determine which institutional sectors have the most negative net worth. Data are retrieved from The Indonesia Financial Account Balance Sheet using Whom-To-Whom matrix that link interaction among the institutional sectors in the financial system. The study selects the most vulnerable institutions to construct the stock network. Then the study analyze its structure and compute the main metrics with the net worth formula [53] as the Vulnerability Indicator:

$${\text{W}}_{\text{i}}=\text{Ai}+\Sigma {\text{V}}_{\text{xi}}-\Sigma {\text{V}}_{\text{ix}}$$ (5)

Whereas W_i_: net worth in the node i, Ai: total asset in node i, and ƩV_xi_ − ƩV_ix_: difference of leverages in and out to the node.

A positive net worth value indicates the value of assets exceeds the liabilities. In contrast, a negative net worth implies that the liabilities exceed their assets [54]. Systemic Risk Indicator uses network centrality i.e., eigenvector centrality (eigen centrality) which is a measure of importance or influence a node has over other nodes in a network [48], as well as the weighted degree and clustering coefficient. The research investigates the weight of each link and its distribution using transition matrix as the weight degree centrality proposed by Candeloro, Savini, and Conte [55]. Watts and Strogatz define clustering coefficient as the number of existing triangles around a node for quantity and describe how the node embedded into the network [56]. This clustering coefficient is important in disclosing instance contagion propagation and a good measure of systemic risk [57].

#### 3.2.3 Difference-in-Differences

The Difference-in-Differences (DID) method is commonly used in impact evaluation [58–60]. The method is later used in research to exercise the time series data of net-worth of six institutional sectors. Comparing the net-worth of each institution in the financial system between pre-Covid period (2015–2019) and during Covid-19 period (2020–2021).

The DID method requires two groups, namely the treatment group and the control group, and a minimum of two observation periods before-after treatment. In this case, the treatment group is the net-worth of institutional sectors affected by the COVID-19 pandemic. Additionally, there is a control group in the net-worth not affected by the pandemic. The characteristics of the treatment group and the control group must be similar. These are available in the banking report provided every month (longitudinal / panel of respondents).

The DID method assumes that parallel trends/slopes do not change (trends over time are the same in both groups). This is such a pseudo experimental design because it is not a real different separated group (treatment and control group) but only separating the data period. The Current Account Balance (CAB) and net-worth data in the research hold the assumption indicated by that quarterly dataset before the Covid-19 period (control group) and during the pandemic period (treatment group) are continuous data series (see Appendix for the detail).

DID estimating formulation, for example in the two-period case, simply estimate the linear regression and the model that contain the following specification [61]:

$$Y_{it}=\alpha +\beta *Treated_{it}+ɣ*Post_{\text{it}}+\delta *{\left(Treated*Post\right)}_{\text{it}}+\varepsilon_{\text{it}}$$ (6)

Here, *Y*_*it*_ is CAB as a dependent variable. *Treated*_*it*_ is net worth as a variable indicating whether a unit is treated. *Post*_*it*_ is a dummy variable indicating the post-treatment period. (Treated * Post)_it_ is an interaction variable, and δ is a Difference in Differences (DID) estimator. Here, there are two hypotheses, namely:

H0: Statistically no difference between pre-Covid and during Covid period
H1: Statistically there is a significant difference between pre-Covid and during Covid period.

#### 3.2.4 Macroeconomic linkages

The last method employed in this research is the macroeconomic linkages (data in S3 Data Macroeconomic Linkages Data), derived from the IMF framework [62]. The study can continue the identity on (3) become more complete information that may be shown as:

S–I=CAB=Useofforeignsaving (7)

Economy wide saving-investment gap Current account balance.

In the broader macroeconomic sense, it reflects the gap between the economy’s disposable income and absorption, or between saving and investment, and foreign savings used by the domestic economy to finance an inflow of foreign resources. This relationship highlights the importance of the current account balance as a central unifying concept linking the domestic economy with the rest of the world (ROW).

Then the equation on (7) can be rewritten to private (real) sector and government (fiscal) sector as follows:

$${\left(\text{S}–\text{I}\right)}_{\text{p}}+{\left(\text{S}–\text{I}\right)}_{\text{g}}=\text{CAB}$$ (8)

where the subscripts p and g refer to the private and government sector, respectively. It suggests that there are important relationships between the saving-investment gap of the private sector, the overall fiscal position of the government sector, and the current account of the balance of payments (external sector). It focuses on the separate roles that the private and government sectors play in a current account imbalance.

Furthermore, the interrelations among the various sectors in the macroeconomic analyzed with the money flows linkages. This divides the world into the domestic economy and the rest of the world (ROW/external sector), can be further refined by disaggregating the domestic economy into three key sectors (the private sector, government, and the monetary sector).

## 4. Results and discussion

This section is calculating and analysing each institutional sector in the financial system. Three analytical methods will lead to investigate the financial system vulnerability and pandemic impact on the financial system of Indonesia in 2015–2021.

### 4.1 Risk profile results

**Fig 3:**
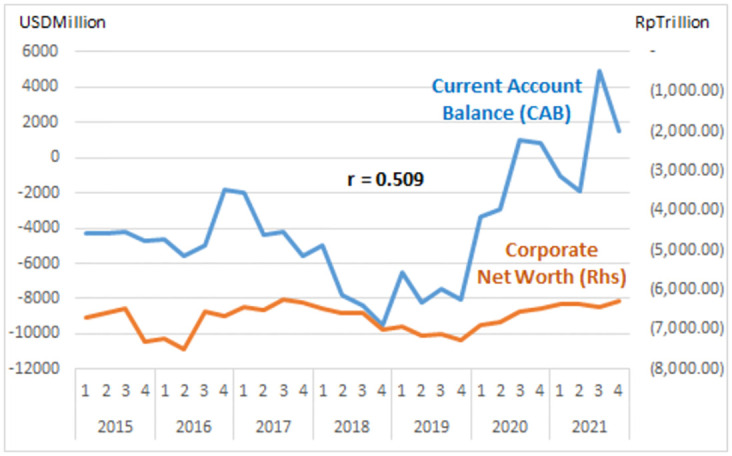
Corporation risk.

**Fig 4:**
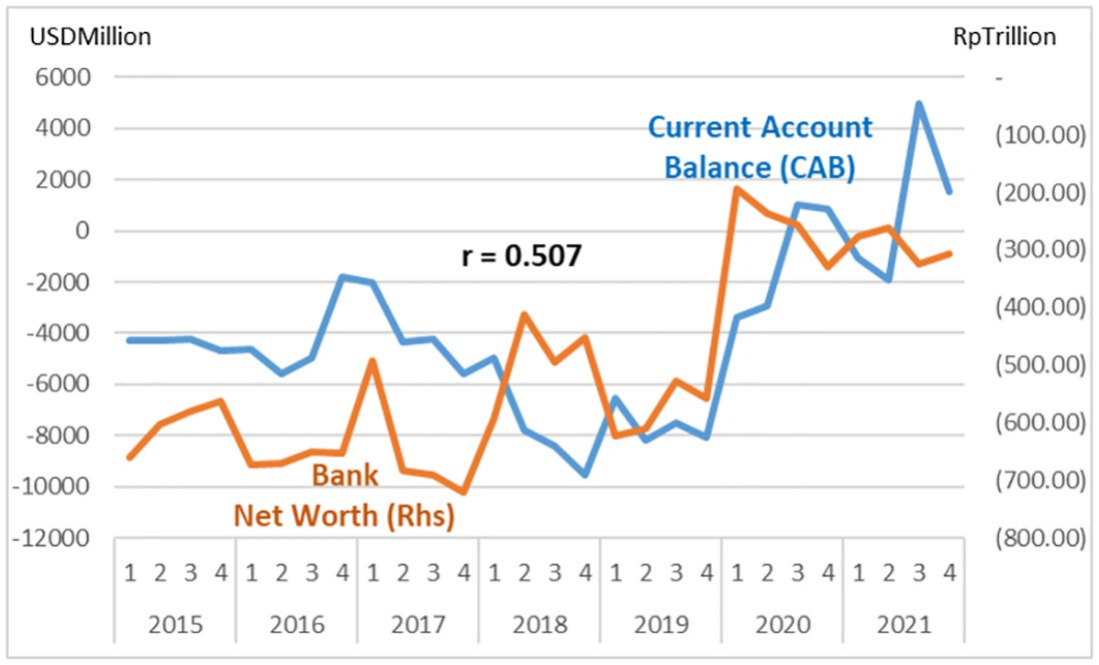
Bank risk.

**Fig 5:**
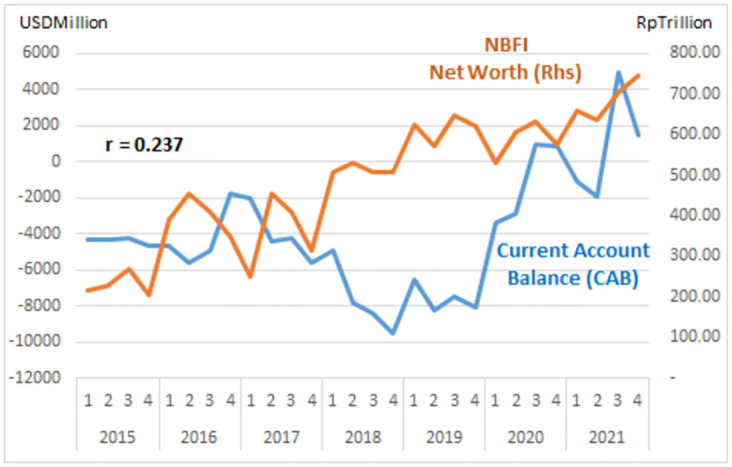
NBFI risk.

**Fig 6:**
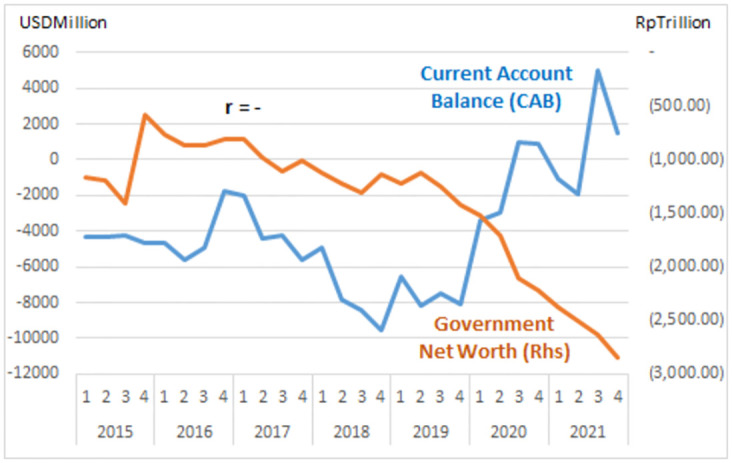
Government risk.

**Fig 7:**
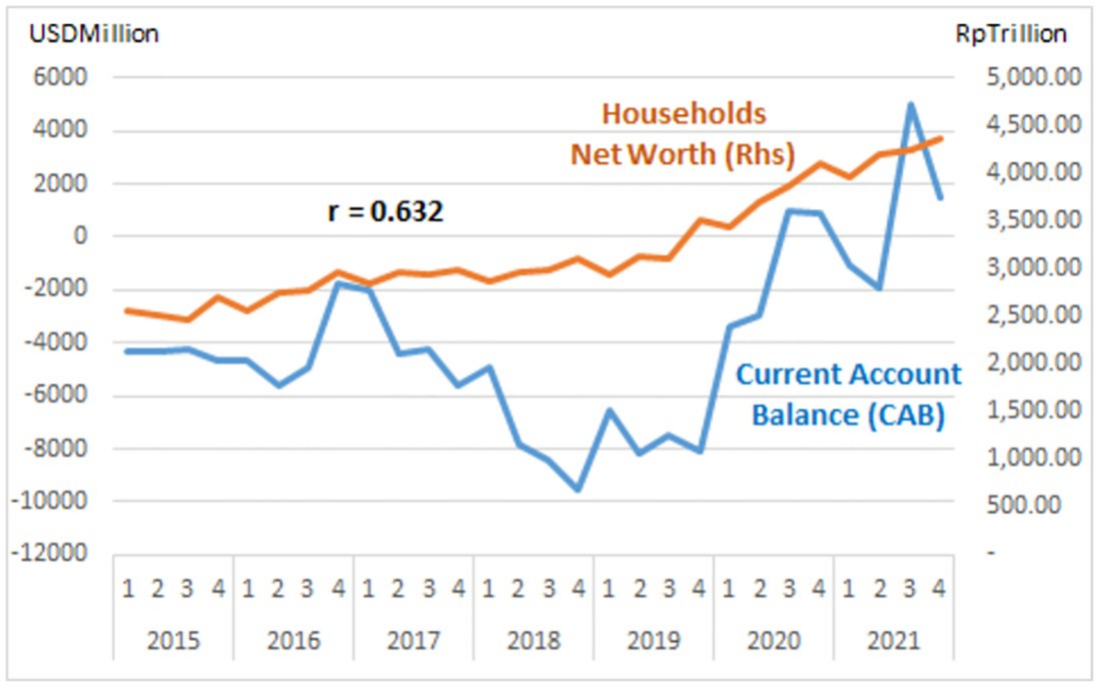
Household risk.

**Fig 8:**
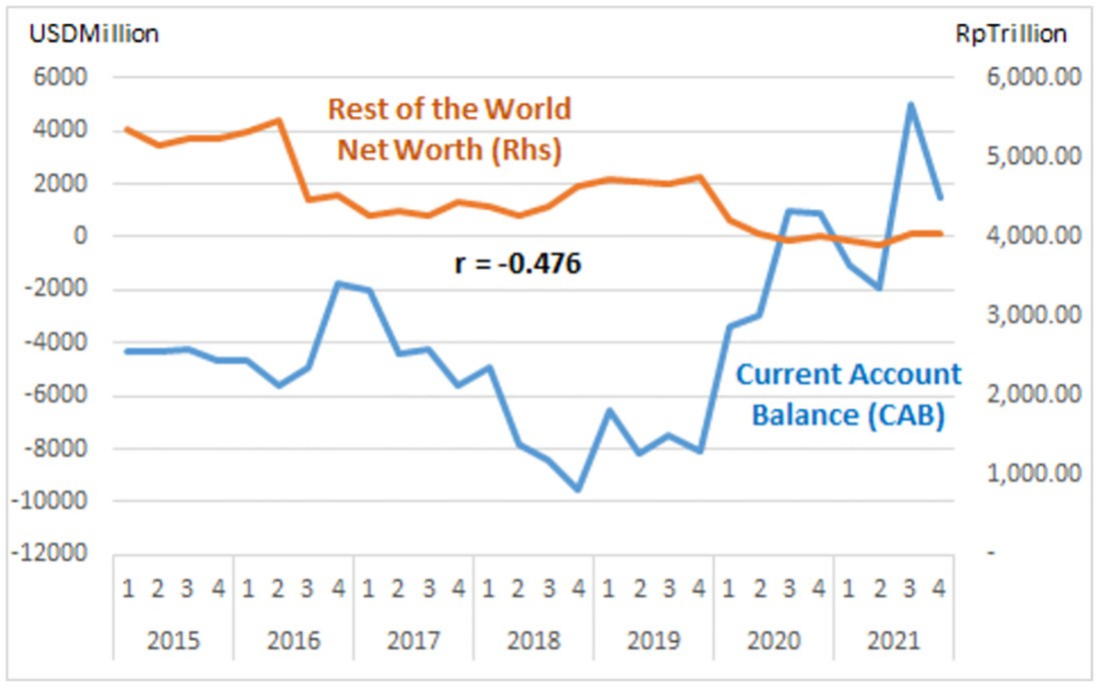
Rest of the world risk.

The study describes graphs of each institutional sector and its relation to the Current Account Balance (CAB) from net-worth series quarterly data (2015: Q1 to 2021: Q4, Table Net-worth in S1 Data Risk Profile and DID Data). The net-worth that indicates the risk can be illustrated in the graph of each institutional risk (see Figs 3–8).

The study observes on the graphs that all institution has quite high correlation with the CAB except NBFI. All institution has positive correlation besides government and the ROW. The study reveals that the financial system is more vulnerable in the pandemic period particularly for banks, government and the ROW indicated by the more declining net-worth (see Figs 3–6).

The risk-analysis results show that the financial system in the net-borrowing conditions in the CAB is minus USD 4billion on average. The condition contributed by the negative net-worth is particularly from corporations, banks, and government, as shown in Table 1.

**Table 1:** Descriptive statistics of the institutional sectors net-worth.

Descr. Stat	CAB	Corp.	Bank	NBFI	Govt	HH	ROW
Mean	-4,019.54	-6704.9	-503.27	484.49	-1,402.33	3,195.67	4,526.64
Stdr. Dev.	3,372.53	352.99	165.92	158.84	614.85	570.77	480.65
Corr (r)	-	0.51	0.51	0.24	-6.67	0.63	-4.48

Notes:

CAB: Current Account Balance (USD million)

Mean: net-worth of each institution (Rp trillion)

Corp.: Non-Financial Corporations (NFCs)

Bank: Other Depository taking Corporations (ODCs)

NBFI: Non-Bank Financial Institutions/ Other Financial Corporations (OFCs)

Govt: Central Government

HH: Households

ROW: Rest of the World/ External Sectors

### 4.2 Network analysis results

For further analysis of the risk profile, the study focusses on the three most negative institutional sectors: corporations, banks, and governments. The time horizon is divided into three consecutive periods that perceived as pre-COVID (2019: Q4) and during COVID (2020: Q4 & 2021: Q4). The data in S2 Data Network Analysis Data.

The Corporation, Government, and Bank have the highest risks in terms of their negative weight (net-worth) in the pre-pandemic era, Quarter IV-2019 (see Tables 2 and 3). Tables 4 and 5 show the weight and weight percentage of each link in the network. The Bank has high liabilities to households, corporations (in terms of deposits), and to the ROW in non-resident equities. Corporation is the most vulnerable institution in the financial system network, due to the highest liabilities to external sectors foreign investment. The government still has high leverage to the ROW in debt securities owned by non-residents and external loans. These conditions are also indicated by the highest weighted degree in those institutional sectors except government.

**Table 2:** Whom-To-Whom matrix in 2019.

Position		Liabilities (Trillion Rp)					
Q4-2019		NFCs	ODCs	OFCs	CG	HH	ROW
**Assets**	**NFCs**	-	1,966.4	151.6	371.3	-	3,054.5
	**ODCs**	2,938.1	-	344.3	777.0	2,688.7	216.8
	**OFCs**	717.8	619.7	-	649.0	362.2	125.5
	**CG**	2,430.7	650.9	128.1	-	90.8	22.6
	**HH**	1,536.0	3,614.8	977.2	225.1	-	-
	**ROW**	5,250.4	1,569.6	266.3	2,786.7	-	-

**Table 3:** Network calculation in 2019.

Institutional Sectors	weight (net-worth)	weighted `indegree	weighted outdegree	weighted degree
Bank (ODCs)	-558	6965	8422	15387
Corporations (NFCs)	-7258	5544	12873	18417
Government (CG)	-1424	3196	4160	7356
ROW	4751	9873	3421	13294
Household (HH)	3514	6353	3142	9495
NBFI (OFCs)	622	1826	1739	3565

**Table 4:** Weighted adjacency matrix of 2019.

Institutional Sectors	Bank	Corp.	Govt.	ROW	HH	NBFI	Sum
Bank (ODCs)		1966	651	1570	3615	620	8422
Corporations (NFCs)	2938		2431	5250	1536	718	12873
Government (CG)	777	371		2787	225		4160
ROW	217	3055	23			126	3421
Household (HH)	2689	0	91			362	3142
NBFI (OFCs)	344	152		266	977		1739

**Table 5:** Transition matrix of 2019.

Institutional Sectors	Bank	Corp.	Govt.	ROW	HH	NBFI
Bank (ODCs)	0.0%	23.3%	7.7%	18.6%	42.9%	7.4%
Corporations (NFCs)	22.8%	0.0%	18.9%	40.8%	11.9%	5.6%
Government (CG)	18.7%	8.9%	0.0%	67.0%	5.4%	0.0%
ROW	6.3%	89.3%	0.7%	0.0%	0.0%	3.7%
Household (HH)	85.6%	0.0%	2.9%	0.0%	0.0%	11.5%
NBFI (OFCs)	19.8%	8.7%	0.0%	15.3%	56.2%	0.0%

Note: NFCs (Non-Financial Corporations = Corporations), ODCs (Other Depository taking Corporations = Banks), OFCs (Other Financial Corporations = Non-Bank Financial Institutions/NBFI), CG (Central Government/Govt.), HH (Household), ROW (Rest of the World).

In Quarter IV-2020, Tables 6 and 7 show the outcome of the pandemic period, where the same condition as the Corporation, Government and Bank presents systemic (vulnerable) risks with respect to negative weight (net worth). Again, Bank is the most important institution in the financial system network indicating by its eigenvector centrality. Corporation is the most vulnerable in the period in terms of its weight. It has a high leverage on the rest of the world, mostly equity and external loans. The government is also vulnerable because of its issued debt securities which the bank bought mainly for the vaccine and social safety net. The highest weighted degree is in those institutional sectors except government (see Tables 8 and 9).

**Table 6:** Whom-To-Whom matrix in 2020.

Closing		Liabilities (Trillion Rp)					
Tw IV-2020		NFCs	ODCs	OFCs	CG	HH	ROW
**Assets**	**NFCs**	-	2,208.4	158.9	391.2	-	3,326.2
	**ODCs**	2,794.7	-	311.4	1,187.0	2,750.9	297.2
	**OFCs**	682.3	660.1	-	638.5	307.9	133.9
	**CG**	2,508.9	742.3	156.3	-	106.0	26.5
	**HH**	1,547.1	3,985.3	1,002.2	359.6	-	-
	**ROW**	5,112.9	1,349.1	234.7	2,917.1	-	-

**Table 7:** Network calculation in 2020.

Institutional Sectors	weight (net-worth)	weighted indegree	weighted outdegree	weighted degree
Bank (ODCs)	-330	7341	8944	16285
Corporations (NFCs)	-6464	6084	12646	18730
Government (CG)	-2225	3384	4855	8239
ROW	4016	9614	3784	13398
Household (HH)	4095	6894	3165	10059
NBFI (OFCs)	575	1784	1707	3491

**Table 8:** Weighted adjacency matrix of 2020.

Institutional Sectors	Bank	Corp.	Govt.	ROW	HH	NBFI	Sum
Bank (ODCs)		2208	742	1349	3985	660	8944
Corporations (NFCs)	2795		2509	5113	1547	682	12646
Government (CG)	1187	391		2917	360		4855
ROW	297	3326	27			134	3784
Household (HH)	2751	0	106			308	3165
NBFI (OFCs)	311	159		235	1002		1707

**Table 9:** Transition matrix of 2020.

Institutional Sectors	Bank	Corp.	Govt.	ROW	HH	NBFI
Bank (ODCs)	0.00%	24.69%	8.30%	15.08%	44.56%	7.38%
Corporations (NFCs)	22.10%	0.00%	19.84%	40.43%	12.23%	5.39%
Government (CG)	24.45%	8.05%	0.00%	60.08%	7.42%	0.00%
ROW	7.85%	87.90%	0.71%	0.00%	0.00%	3.54%
Household (HH)	86.92%	0.00%	3.35%	0.00%	0.00%	9.73%
NBFI (OFCs)	18.22%	9.31%	0.00%	13.77%	58.70%	0.00%

As shown in Tables 10 and 11, the Bank is the most important institution in the financial system network even with slightly minus net worth particularly at the end of pandemic on the Quarter IV-2021. The Bank has the highest leverage to households and corporations related to their third-party funds (deposits). Corporation and Government have systemic risks in terms of high negative weight (net worth) indicating the vulnerability in those two institutions. They have big leverage to ROW and bank. Once again, the highest weighted degree is in those institutional sectors except government (see Tables 12 and 13).

**Table 10:** Whom-To-Whom matrix in 2021.

Closing		Liabilities (Trillion Rp)					
Tw IV-2021		NFCs	ODCs	OFCs	CG	HH	ROW
**Assets**	**NFCs**	-	2,745.7	160.4	383.7	-	3,620.1
	**ODCs**	2,872.6	-	339.5	1,488.0	2,930.6	276.2
	**OFCs**	694.8	758.5	-	779.0	300.7	155.2
	**CG**	2,717.7	795.3	144.3	-	138.5	29.0
	**HH**	1,585.6	4,172.1	1,073.3	543.6	-	-
	**ROW**	5,501.6	1,349.7	238.7	2,861.6	-	-

**Table 11:** Network calculation in 2021.

Institutional Sectors	weight (net-worth)	weighted indegree	weighted outdegree	weighted degree
Bank (ODCs)	-306	7908	9867	17775
Corporations (NFCs)	-6284	6910	13374	20284
Government (CG)	-2845	3681	5278	8959
ROW	4040	9998	4080	14078
Household (HH)	4360	7375	3371	10746
NBFI (OFCs)	748	1910	1812	3722

**Table 12:** Weighted adjacency matrix of 2021.

Institutional Sectors	Bank	Corp.	Govt.	ROW	HH	NBFI	Sum
Bank (ODCs)		2746	795	1395	4172	759	9867
Corporations (NFCs)	2873		2718	5502	1586	695	13374
Government (CG)	1488	384		2862	544		5278
ROW	276	3620	29			155	4080
Household (HH)	2931	0	139			301	3371
NBFI (OFCs)	340	160		239	1073		1812

**Table 13:** Transition matrix of 2021.

Institutional Sectors	Bank	Corp.	Govt.	ROW	HH	NBFI
Bank (ODCs)	0.0%	27.8%	8.1%	14.1%	42.3%	7.7%
Corporations (NFCs)	21.5%	0.0%	20.3%	41.1%	11.9%	5.2%
Government (CG)	28.2%	7.3%	0.0%	54.2%	10.3%	0.0%
ROW	6.8%	88.7%	0.7%	0.0%	0.0%	3.8%
Household (HH)	86.9%	0.0%	4.1%	0.0%	0.0%	8.9%
NBFI (OFCs)	18.8%	8.8%	0.0%	13.2%	59.2%	0.0%

**Fig 9:**
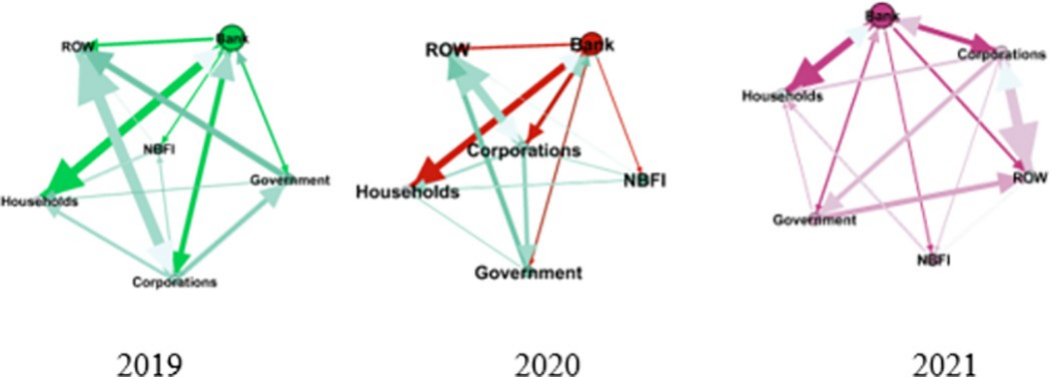
Network of financial system Pre-COVID (2019) and during COVID (2020–2021).

The three consecutive period networks depicted in Fig 9 and the network analysis resumed in Tables 14 and 15.

**Table 14:** Eigen centrality and clustering 2019–2021.

Institutional Sectors	clustering	eigen centrality
Bank (ODCs)	0.75	1
Corporations (NFCs)	0.8	0.83841
Government (CG)	0.75	0.83841
ROW	0.83	0.83841
Household (HH)	0.83	0.83841
NBFI (OFCs)	0.75	0.83841

**Table 15:** Network analysis resume.

Institutional Sectors	Bank	Corp.	Govt.	ROW	HH	NBFI
Bank (ODCs)		increase	fluctuate	decrease	fluctuate	increase
Corporations (NFCs)	decrease		increase	increase	fluctuate	decrease
Government (CG)	increase	decrease		decrease	increase	
ROW	fluctuate	fluctuate	steady			fluctuate
Household (HH)	increase		increase			decrease
NBFI (OFCs)	fluctuate	fluctuate		decrease	increase	

It is obvious that the topology of the financial system network has no change in three consecutive years. This means that there is no changing in terms of relationship between institutions. As shown in Fig 9, Bank becomes a central of network and has the biggest linkages with household and corporation. It confirms that the two institutions act as the banks’ main depositors and acquire much more credit deliveries.

Bank is also the highest eigenvector centrality meaning that it is the most important or influential institution to others in the financial system network. The situation is similar before and during COVID-19 pandemic (see Table 14). Clustering coefficient, introduced by Watts and Strogatz [63], refers to the number of existing triangles around a node respect to the number of potential ones, providing a quantity suitable to account for how the node embedded into the structure, in terms of connections. In this case, ROW, Households and Corporations have clustering coefficient score around 0.8, and more embedded to the network compared to others. Thus, it is easier to be affected by the condition. The clustering indicates vulnerability in systemic risk [56] so that those three institutions become the most vulnerable in Indonesia’s financial system.

The number of significant links connected to each node based on the percentage weight (transition matrix) from 2019 to 2021. Finally, we can resume for each connection between two nodes of each institutional sector by looking at the percentage weight changing which above 20% in yellow highlight cells presumed more than a fifth liabilities of one institution hold to others (see Table 15).

**Fig 10:**
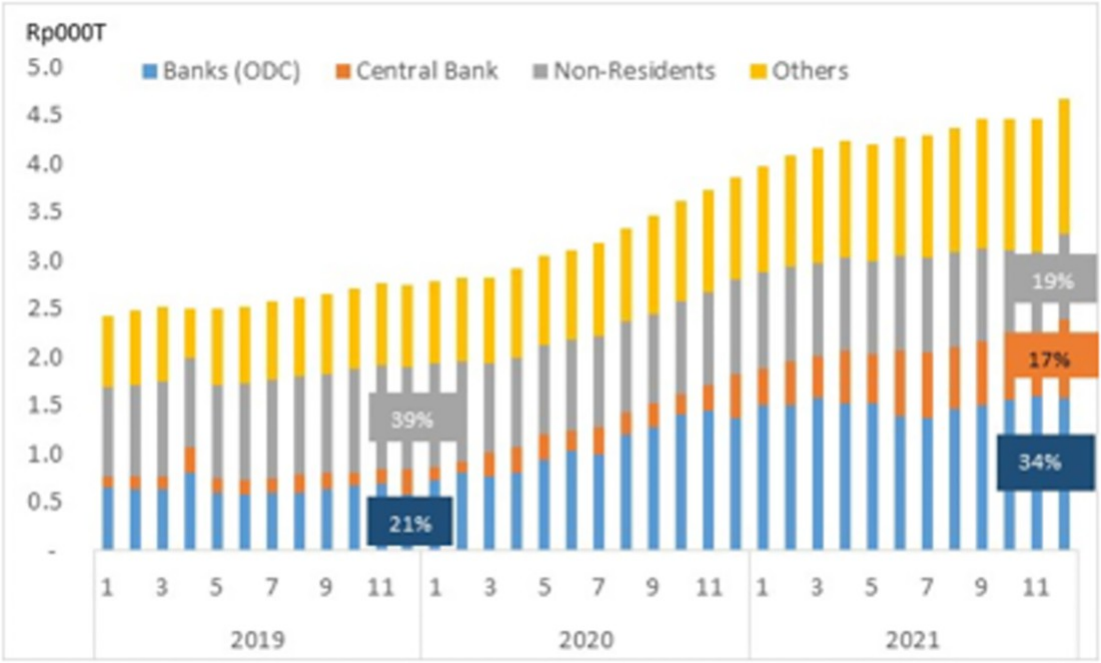
Government bonds ownership.

One institution that increased its liabilities during the COVID-19 period is the government. In the pandemic situation, the Indonesia government sought to avoid negative health & social impacts of the COVID-19 on the people with some policy interventions. They distributed vaccine freely to all citizens to prevent virus spreading and gave a social safety net for helping household economic. All costs have been burdening the fiscal budget so that financed by issuing the public obligations that much bought by the banking sectors and the central bank. The banks (ODC) and central bank have about a half of government bonds ownership at the end 2021, each Rp1.6 thousand trillion and Rp800 trillion, respectively (Ministry of Finance, 2022). It was almost double fold in percentage compared to their ownership at end of year before the COVID-19 pandemic (see Fig 10). Fortunately, the condition has decreased government bonds depending on the Rest of the World (ROW) indicated by a half curbing of non-residents ownership percentage.

### 4.3 Empirical results

In the regression, the study set that the dependent variable *Y*_*t*_ is the Current Account Balance (CAB); the independent variable *X*_*t*_*Inst*.*sector*_ represents net-worth of each institutional sector; *D*_*t*_*Covid*_ is a dummy variable that takes the value of one for the quarterly period from 2020:Q1 to 2021:Q4 (the COVID-19 pandemic period), and zero otherwise; and *X*_*t*_*Inst*.*sector*_ *D*_*t*_*Covid*_ is the interaction variable, data in S1 Data Risk Profile and DID Data. The model has the following specifications for each institutional sector is:

$$Y_{t}=\alpha +\beta \;X_{t\_Inst\_sector}+ɣD_{t\_Covid}+\delta \;X_{t\_Inst\_sector}\;D_{t\_Covid}$$ (9)

Thus, the model specification for corporation as a sample for another institutional sector becomes:

$$Y_{t}=\alpha +\beta \;X_{t\_Corp}+ɣD_{t\_Covid}+\delta \;X_{t\_Corp}\;D_{t\_Covid}$$ (10)

The estimation result shows that the interaction variable of corporation *X*_*t*_*Corp*_ *D*_*t*_*Covid*_ has a coefficient of 4.89, statistically insignificant at the 5% significance level. Here the corporation net-worth underwent no significant change during the pandemic period in 2020–2021 from the pre-COVID-19 period (2015–2019). Oppositely, the estimation result for bank has -37.03 of the interaction variable *X*_*t*_*Bank*_ *D*_*t*_*Covid*_, statistically significant at the 5% significance level. This result reveals that net-worth of bank experienced a significant change during the pandemic period rather than of the previous period. It causes the 2020–2021 regression coefficient (gradient) of *X*_*t*_*Bank*_ become -48.96, much lower than the -11.94 from the pre-COVID-19 period. The calculation results of DID for each institutional sectors are shown in Tables 16–21.

**Table 16:** Corporation DID result.

Dependent Variables: CAB	A. pre-COVID period 2015: Q1–2019: Q4	B. COVID period 2020: Q1–2021: Q4
Independent Variables:	Coefficient (p-value)	Coefficient (p-value)
Corp	2.1829 (0.107)	2.1829 (0.107)
Covid	-	36724.84 (0.157)
CorpCovid	-	4.8859 (0.215)
Constant	9221.057 (0.308)	9221.057 (0.308)
N	20	8

**Table 17:** Bank DID result.

Dependent Variables: CAB	A. pre-COVID period 2015: Q1–2019: Q4	B. COVID period 2020: Q1–2021: Q4
Independent Variables:	Coefficient (p-value)	Coefficient (p-value)
Bank	-11.9384* (0.025)	-11.9384* (0.025)
Covid	-	-827.2126 (0.874)
BankCovid	-	4.8859 (0.215)
Constant	-12684.61* (0.000)	-12684.61* (0.000)
N	20	8

**Table 18:** NBFI DID result.

Dependent Variables: CAB	A. pre-COVID period 2015: Q1–2019: Q4	B. COVID period 2020: Q1–2021: Q4
Independent Variables:	Coefficient (p-value)	Coefficient (p-value)
NBFI	-10.5015* (0.001)	-10.5015* (0.001)
Covid	-	-15519.99* (0.018)
NBFICovid	-	36.4733* (0.001)
Constant	-1129.073 (0.368)	-1129.073 (0.368)
N	20	8

**Table 19:** Government DID result.

Dependent Variables: CAB	A. pre-COVID period 2015: Q1–2019: Q4	B. COVID period 2020: Q1–2021: Q4
Independent Variables:	Coefficient (p-value)	Coefficient (p-value)
Govt	4.9729* (0.017)	4.9729* (0.017)
Covid	-	-9322.18* (0.037)
GovtCovid	-	-9.2035* (0.001)
Constant	-268.0468 (0.901)	-268.0468 (0.901)
N	20	8

**Table 20:** Household DID result.

Dependent Variables: CAB	A. pre-COVID period 2015: Q1–2019: Q4	B. COVID period 2020: Q1–2021: Q4
Independent Variables:	Coefficient (p-value)	Coefficient (p-value)
HH	4.7703* (0.011)	4.7703* (0.011)
Covid	-	-32926.02* (0.004)
HHCovid	-	10.9567* (0.001)
Constant	8162.225 (0.115)	8162.225 (0.115)
N	20	8

**Table 21:** ROW DID result.

Dependent Variables: CAB	A. pre-COVID period 2015: Q1–2019: Q4	B. COVID period 2020: Q1–2021: Q4
Independent Variables:	Coefficient (p-value)	Coefficient (p-value)
ROW	0.5434 (0.681)	0.5434 (0.681)
Covid	-	32981.97 (0.418)
ROWCovid	-	-6.7556 (0.502)
Constant	-8149.616 (0.201)	-8149.616 (0.201)
N	20	8

Comparing the net-worth for each institution indicates vulnerability in the financial system between pre-Covid period (2015–2019) and during the Covid-19 period (2020–2021). Table 22 shows the DID result which reveals that vulnerability of Corporations and Rest of The World are statistically no different between pre-Covid and during Covid period. Meanwhile, vulnerability of Banks, NBFIs, Government, Households are significantly different between pre-Covid and during Covid period.

**Table 22:** DID resume.

Variables	Corp	Bank	NBFI	Govt.	HH	ROW
Interaction Coefficients	4.886	-37.033	36.473	-9.204	10.957	-6.756
Significant at 5%	x	√	√	√	√	x

The insignificant DID result for corporation emerges a further investigation on the sub-industrial sectors. Subsequently, the study calculates the DID process for 16 sub-industrial sectors (see Table 23). The result shows that even though for corporation is indifferent, many more industries have significant difference performances during the pandemic than those of previous periods, such as food and beverages, tobacco, paper and publisher, chemistry and pharmacy, transport vehicles, et cetera according to GDP nominal calculation based. However, the calculation using GDP growth based only shows significant results for textile and electronic sub industries.

**Table 23:** DID result for industrial subsectors.

Sub-Industries	DID Results	
	GDP growth	GDP nominal
Coal & Oil Refining	x	x
Food & Beverages	x	√
Tobacco	x	√
Textile	√	x
Leather and Footwear	x	x
Wood, Bamboo, Rattan	x	x
Paper & Publisher	x	√
Chemistry & Pharmacy	x	√
Rubber & Plastic	x	x
Non-Metal Mining	x	√
Basic Metal	x	√
Electrical, Computer & Optic	√	x
Machine & Apparatus	x	√
Transport Vehicle	x	√
Furniture	x	√
Others & Services	x	√

√: significant at 5% level, and x otherwise

The study uses the GDP nominal data at current prices for DID sub-sector industries calculation. Some explanations might be revealed from several sources. The food and beverage industry consistently makes a significant contribution to national economic growth. Even during pressure from the impact of the Covid-19 pandemic, the productivity of these sub-sector industries remains maintained. The food and beverage industry are also a sector that has high demand during the pandemic. This is because people still need to consume nutritional intake to increase their body’s immunity to maintain health [64].

Furthermore, the contribution of consumer spending to buy Fast Moving Consumer Goods (FMCG) was also quite large, namely up to 12% at the end of 2021. Its position is third after saving and paying off debt (21%) and vacations (13%). FMCG is a product that has a fast turnover at a low cost. These products usually have a relatively short shelf life considering that they spoil more quickly. Some of the FMCG products include instant food and packaged beverages.

Concerning the tobacco industry conditions during the pandemic is that cigarette production tends to increase, adjusting to market demand. This is probably due to government policies for implementing restrictions on community activities that make people work from home or have limited activities outside the home, so that smokers have much more opportunities to smoke [65].

Economic pressure during the pandemic did not influence negatively on cigarette consumption, even scale up. Results for the national economic & social survey in the last 3 years noted that the average per capita expenditure per month for cigarettes and tobacco commodities was in the second highest place after basic needs for processed food and beverages. A survey conducted by IDEAS on 1,013 heads of poor families in Greater Jakarta, Greater Semarang, Greater Surabaya, Greater Medan, and Greater Makassar in January—February 2021 found 77.1% said their consumption of cigarettes had not decreased during the pandemic. Even increased. The crisis has not made poor families reduce the burden of smoking cigarettes. The income and purchasing power of smokers as well as the price and availability of cigarettes are the main factors that facilitate individual cigarette consumption. As many as 73.2% of poor smokers continue to spend on cigarettes despite declining economic conditions, meaning that expenses for other necessities have decreased or even eliminated so they can continue smoking in the same quantity [66].

During the COVID-19 pandemic, the printing industry in Indonesia showed a significant decline. This can be seen in the market figures for the printing industry, which fell -49.10% from the previous quarter and -48.23% compared to the previous year. Most micro and small business actors admit their sales have decreased compared to pre-pandemic sales. Some printing industries have gone out of business and laid off employees temporarily.

The COVID-19 pandemic has had a profound impact on the printing industry. This is because income is running low, production is disrupted, and machine installments cannot be paid. However, fixed expenses such as employee salaries must be paid. This is what puts the printing industry on the verge of bankruptcy and even must close its business. In addition, many printing industries are experiencing financial difficulties because of many orders that have been executed but delayed payments and experienced cancellations. The printing industry experienced a decline in turnover and a decline in production of more than 70%. As a result of these unfavorable conditions, many printing industries have started laying off employees without pay or only paying half the salary, schedule to work in shifts, and releasing employees with daily status.

The COVID-19 outbreak has created opportunities for pharmaceutical production. However, due to dependency on imported raw materials approximately 60 percent of it is imported from China, then the pandemic reduced production of the Indonesian pharmaceutical industry up to 60 percent in May 2020. The positive effect of the Covid-19 pandemic for the pharmaceutical industry is the relaxation of the regulation that helps the pharmaceutical industry.

The COVID-19 pandemic which began early 2020 made the need for vitamins, supplements and herbal medicine for stronger metabolism increased, so that the pharmaceutical industry playing in this sector receive large growth, marked by the Industrial GDP for Chemistry, Pharmacy and Traditional Medicine which grew the tallest of the 15.3 (fifteen) Industrial groups Non-oil and Gas Processing in 2020, approximately reaching 9.39% (YoY), growth is also increasing compared to 2019, at 8.48% (YoY). Chemical Industry Contribution, Pharmacy and Traditional Medicine also increase in 2020 by 10.75% to GDP of Processing Industry Non-oil rather than contribution of 9.56% in 2019. Throughout 2020, demand for pharmaceutical commodities and medical devices experienced a significant increase as response from society and government to anticipate and overcome COVID-19. Enhanced at the highest sales are personal protective commodity by 50.3% from previously only by 0.1%. While the increase in greatest demand for health commodities are for a mask of 12.6%, 3.1% hand sanitizer and hand soap 2.1%. Nevertheless, the sub-sectoral industry condition faces scarred effects from the pandemic [67].

**Fig 11:**
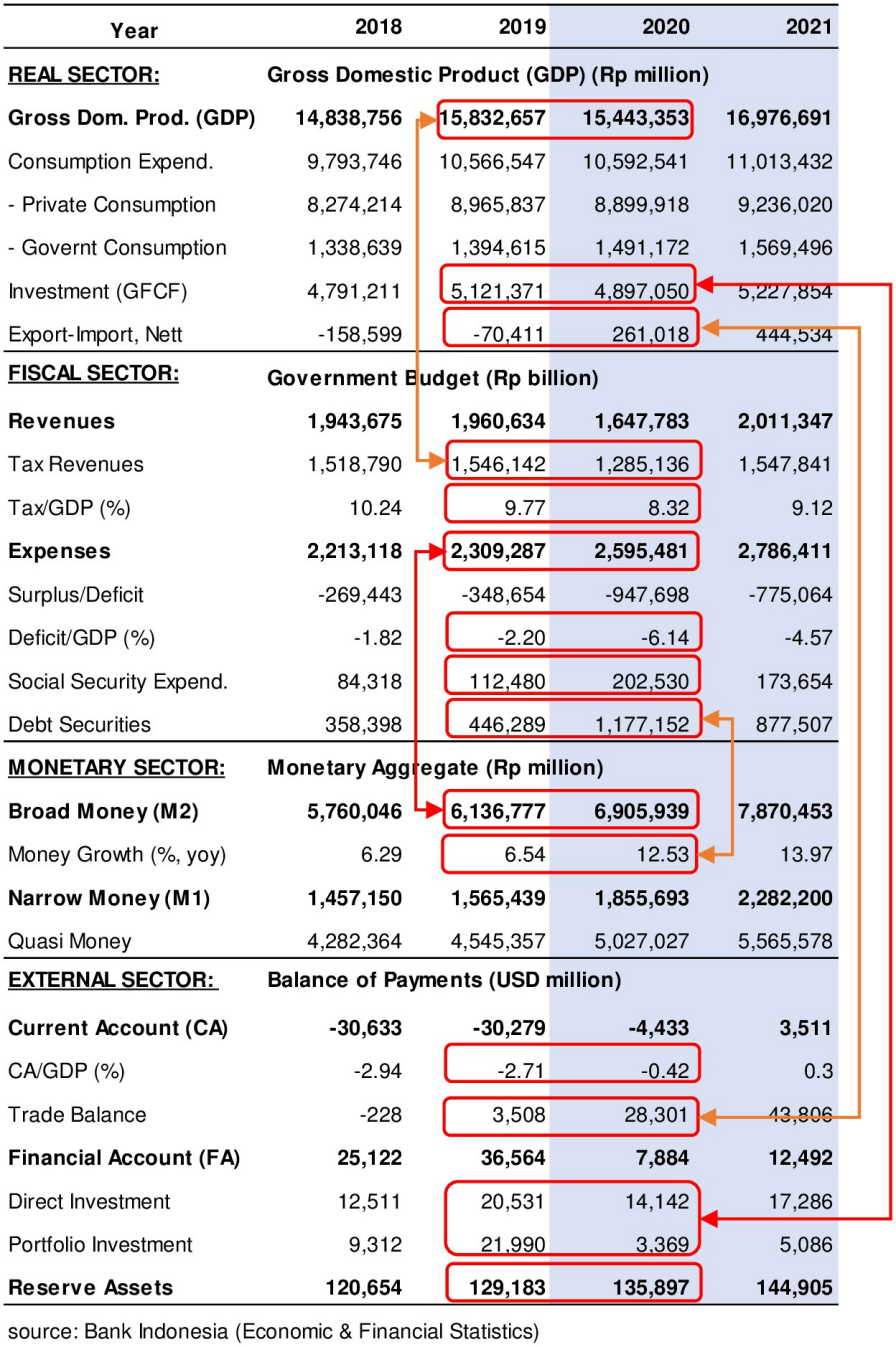
The Indonesia macroeconomic linkages (Summary).

Furthermore, the IMF framework of the macroeconomic linkages can be used to explore what happened to Indonesia’s financial system before and during the pandemic (see Fig 11). On the 2018–2019, before COVID-19, the condition remained stable highlighted by several indicators such as Gross Domestic Product (GDP) growth positive in the real sector, government revenues add in the fiscal sector, money growth was quite stable and current account deficit big enough with high foreign investment in external sector. Notwithstanding, Qadri et al. [68] find that foreign direct investment (FDI) has a significant negative relationship with environmental degradation in Pakistan. Conversely, the green finance has a significant positive relationship with environmental restoration. Likewise, a psychological green climate has stimulated employees of corporations to perform pro-environmental behaviors for better environmental performance [69].

Compared to the situation during COVID-19 period, the macroeconomic condition was descriptively changed, see Fig 11. The declining GDP due to freezing economy activities caused decrease of tax revenues in the government budget. Those are indicated by tax upon GDP ratio that decline from 9.77% to 8.32% even though rebound to 9.12% in 2021. Meanwhile, government expenditures spike mostly for social security funds included vaccines procurement and socio-economic safety net as national economic recovery programs for the poor and micro-small enterprises achieved approximately Rp405 trillion (Ministry of Finance, 2020). This is stated in Law Number 2 of 2020 concerning State Financial Policy and Financial System Stability for Handling the 2019 Corona Virus Disease (COVID-19) Pandemic and/or in the Context of Dealing with Threats that Endanger the National Economy and/or Financial System Stability. Thus, the fiscal deficit increased from -2.2% to -6.14% beyond the rule at 3.0% of GDP and remain high deficit -4.57% in 2021.

The higher government expenditures during the pandemic have increased money growth of 12.53% in the financial system, almost double than in the normal period. The source of those expenditures came from government debt securities issued at amount Rp1177 trillion, almost triple than issued in 2019 and still abundant in 2021 at Rp887 trillion.

### 4.4 Discussion

Hereafter, the external sector shows that during COVID-19 period the current account deficit (CAD) became lower mostly due to the trade balance becoming higher than previously and continues in 2021. The higher trade balance was particularly due to the decreasing import than export performances which relates to the pandemic and scarring affect industries in several countries. Meanwhile, foreign investments in terms of long-terms investment (Direct Investments) and short-terms (Portfolio Investments), are both curb due to the uncertainty in the global money market during the pandemic. These conditions affect the net export-import and investment performance in the real sector.

Compared with other relevant research such as Ma *et al*. [13] who employ structural equation model (SEM) to study the corresponding fast recovery of Small Medium Enterprises (SMEs) in Beijing, China. They discover government policies can significantly reduce the negative impact of the pandemic then suggest the targeted issuance of consumption vouchers and the reduction of administrative barriers. Compared to this study result, the study suggests accelerating the speed recovery, the government need to combine the social safety program for the poor with the SMEs retail that provide basic consumption needs. This will simultaneously impact on the quality of the poor and the SMEs’ performances. There should be well prepared with good quality of data about the poor and the SMEs updated by name and address.

Meanwhile, Hu *et al*. [70] claim that the impact of 2008’s GFC on the economy is more cyclical in the long-term, while the COVID-19 pandemic is a short-term shock with a relatively short oscillation cycle. Furthermore, the economic impact of COVID-19 will not spread into a financial crisis for China and believe that the pandemic is more a health event than an economic crisis for Chinese economy. In contrary, Gunay and Can [71] find that the pandemic induced a more severe contagious effect and risk transmission than the GFC and suggest the presence of strong co-movements of world stock markets with the US equity market, especially in periods of financial turmoil. Our result shows that during pandemic (2020–2021) the vulnerability of Banks, NBFIs, Government, Households increase but nothing to do with Corporations and Rest of The World (ROW). The resilient of corporations might relate to firm size as supported by Khan et al [72] that find evidence from listed non-financial companies in Pakistan there is negative and significant effect of firm structure, leverage, and networking of capital on corporate cash holding, otherwise for firm size.

In terms of result by sector, Wang *et al*. [73] realize although the pandemic had an overall significant and negative impact on the stock prices of firms listed on SSE, SZSE and ChiNext, such impact seems to be heterogeneous across industries. This affects listed firms in industries such as pharmaceutical and telecommunications positively, while firms from services industries including accommodation, catering, and commercial services negatively. Compared with our results, the impact is similar for corporations. Some industrial sectors remain unchanged such as textile product and footwear whereas others changed, for example food & beverages and electronic devices.

Chen et al. [74] investigate how rural households are suffering from COVID-19 using three loss measures to comprehensively assess the impact of logistics disruptions on rural households’ losses and provide evidence on the micro level of rural households. Our research uses institutional sectors in the financial system on a macroeconomic level result that households were more vulnerable during COVID-19 periods than that of pre-COVID included government, bank, and NBFI, but not for the corporation and the rest of the world. Likewise, it confirmed the private consumption declined in the real sector during the pandemic period.

Meanwhile, Bao and Huang [75] showed that during the COVID-19 pandemic, FinTech firms were more likely to extend credit to new and financially constrained borrowers. Compared to our result in this research that bank and NBFI were more vulnerable during COVID-19 period. Another research [61] found that the banking credit performance in Indonesia financial systems declines during the pandemic amid the economic downturn compared to the pre-COVID-19 period.

## 5. Conclusion

In summary, the study has investigated the financial system vulnerability and pandemic impact on the financial system of Indonesia in 2015–2021. The Financial Account Balance Sheet was exercised to measure risks using risk profile and network analysis. The study reveals that the financial system is more vulnerable in the pandemic period particularly for Banks, Corporations and Government indicated by the most declining net-worth. However, the network analysis shows that in terms of its sector relationship, network structure has not changed in three consecutive years (2019–2021). The network calculate that the ROW, Households and Corporations have the highest clustering coefficient meaning they are the most vulnerable in Indonesia’s financial system.

Furthermore, Difference-in-Differences (DID) method exercises the COVID-19 impact on the financial system. The result is the financial system is more vulnerable during the pandemic than that of previous periods, alongside the external balance slowdown. Nevertheless, the estimation result shows that during the pandemic (2020–2021) the vulnerability of Banks, NBFIs, Government, Households increase but nothing to do with Corporations and the Rest of The World (ROW). Moreover, DID tests on key industry subsectors yielded mixed results. Some key industrial sub-sectors remain stable such as textile products and footwear where others changed, including food and beverages, and electronic devices.

Lastly, the macroeconomic linkages analysis forward that the declining of Indonesia real sectors during the COVID-19 pandemic are affected in some part by the external sectors’ condition. The government expenditures rise to avoid pandemic impact on domestic socio-economic financed with more debt securities issued. This caused money growth in the financial system during the pandemic period than that previously. The contribution appears in examining the impact of the pandemic to the financial system and propose course of actions to overcome this condition. For example, some policy recommendations as courses of actions to mitigate the possible risks to the authorities, such as Government and Central Bank.

This research focuses on financial system vulnerability for institutional sectors based on the Balance Sheet Approach theory. The research does not discuss part of financial system about financial market and financial infrastructure that might influence the vulnerability in the financial system. As future works, the research will be conducted about scarring effects of COVID-19 on several key industrial sub-sectors as well as how to avoid or minimize these negative effects to the financial system.

## Supporting information

S1 Data Dataset for risk profile analysis and DID method. (XLSX)

S2 Data Dataset for network analysis. (XLSX)

S3 Data Dataset for macroeconomic linkages. (XLSX)
